# Clinical Experience of a Novel Optical Coherence Tomography-Guided Coronary Chronic Total Occlusion Re-Entry Device

**DOI:** 10.1016/j.jaccas.2023.102041

**Published:** 2023-10-06

**Authors:** Pratik B. Sandesara, Gregory C. Robertson, Kin F. Chan, Douglas Rowe, Adrian Ebner, Laura Minarsch, William Lombardi, David E. Kandzari, Tomoaki Hinohara, John B. Simpson

**Affiliations:** a Division of Cardiology, Department of Medicine, Emory University School of Medicine, Atlanta, Georgia, USA; b Simpson Interventions, Inc., Campbell, California, USA; c Sanatorio Italiano, Asunción, Paraguay; d MMC Medical, Laguna Beach, California, USA; e Division of Cardiology, University of Washington, Seattle, Washington, USA; f Piedmont Heart Institute, Atlanta, Georgia, USA; g Sequoia Hospital, Redwood City, California, USA

**Keywords:** antegrade dissection and re-entry, catheter, chronic total occlusion, guidewire, image augmentation, image-guided intervention, intravascular imaging, optical coherence tomography, percutaneous coronary intervention

## Abstract

We demonstrated a first-in-human case of successful antegrade dissection and re-entry using an image-guided re-entry catheter that enables real-time high-resolution visualization with graphical augmentation, and precision steering and advancement of a guidewire. The total time from over-the-wire deployment in the proximity of the distal cap to successful re-entry was <20 minutes. (**Level of Difficulty: Advanced.**)

## History of Presentation

A 74-year-old man presented with Canadian Cardiovascular Society class III angina despite receiving optimal medical therapy, including 2 antiangina medications in the setting of chronic total occlusion (CTO) of the left anterior descending artery (LAD).

**Learning Objectives**

• To demonstrate the potential for ADR success and enhanced procedural safety having simultaneous real-time image guidance and precision steering and advancement of a guidewire with the novel image-guided re-entry catheter.
• To appreciate the importance of identifying and avoiding the adventitia and perivascular structure to minimize the risk of perforation.
• To appreciate the precision and high probability of success in re-entry with the visualization and differentiation of the adventitia/perivascular structure and the pulsating true lumen.

## Medical History

The patient had a medical history of coronary artery disease with myocardial infarction 18 months prior to presentation. Additionally, he had a history of smoking, hypertension, hyperlipidemia, and hypothyroidism.

## Differential Diagnosis

The differential diagnosis for the presentation of typical angina in this patient with the aforementioned risk factors included obstructive coronary artery disease, coronary microvascular dysfunction, spontaneous coronary artery dissection, and coronary vasospasm.

## Investigations

**Figure 1:**
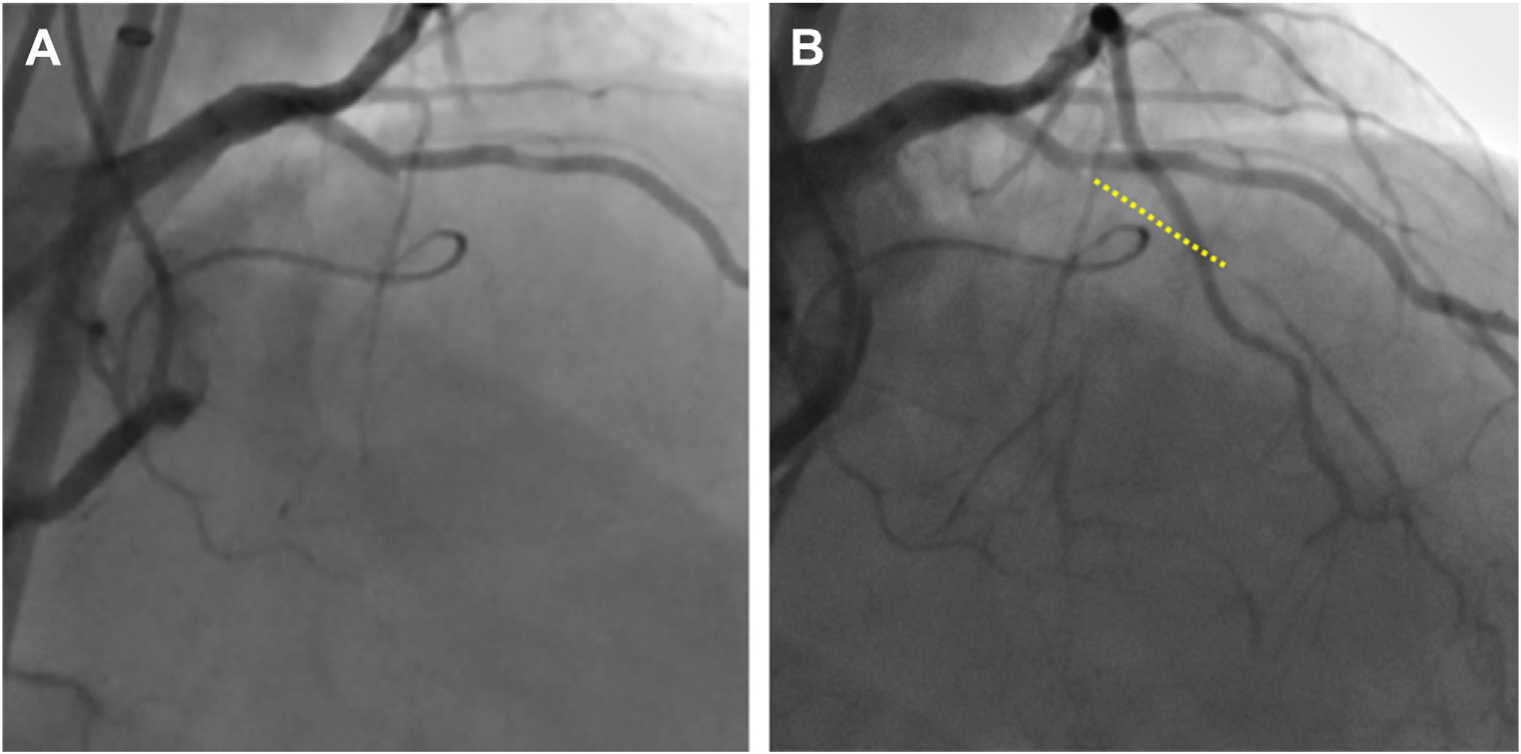
Angiographic View (A) Confirmation of chronic total occlusion (CTO) of the left anterior descending coronary artery with a tapered proximal cap adjacent to the diagonal branch. (B) CTO length is >20 mm with calcification within the CTO segment (yellow dotted line).

Dual coronary angiography showed a mid-LAD CTO with tapered proximal cap (Figure 1A), occlusion length of >20 mm, and calcification within the CTO segment (J-CTO score of 2) (Figure 1B, Video 1).

## Management

**Figure 2:**
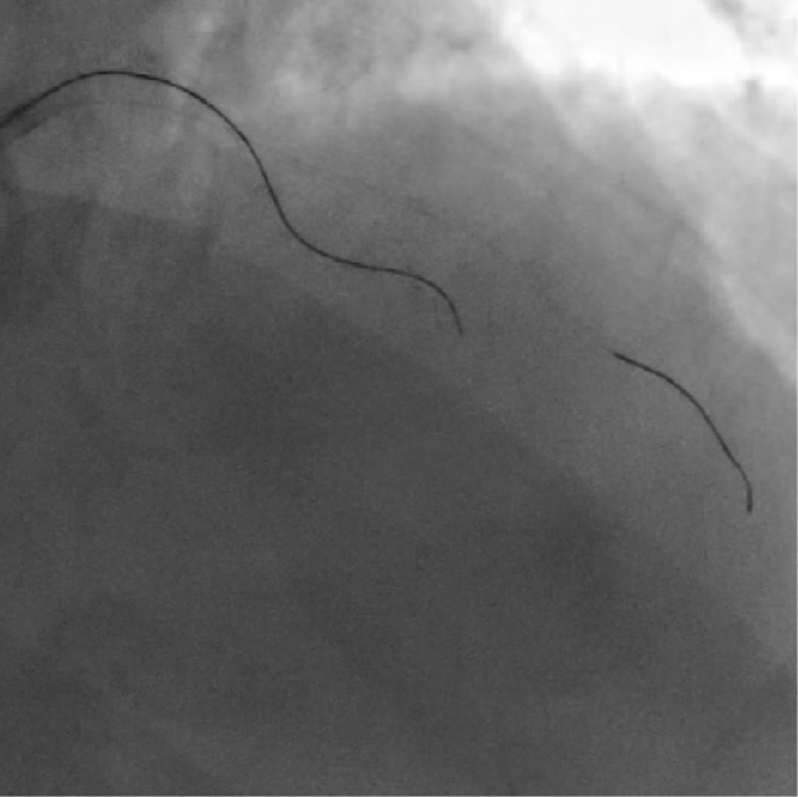
Angiographic View Antegrade wire escalation was initially attempted for approximately 45 minutes without success.

**Figure 3:**
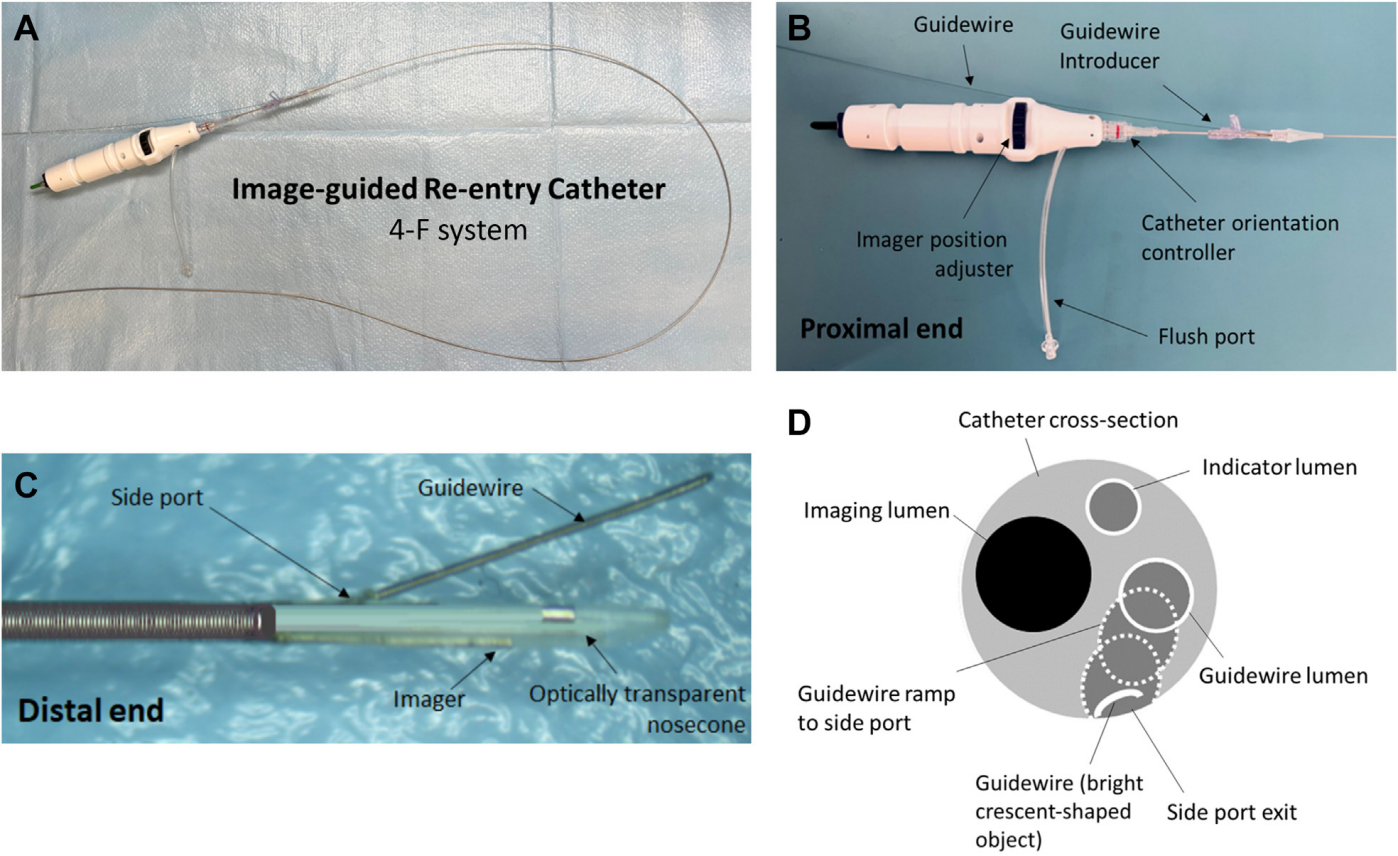
A 4-F Image-Guided Re-entry Catheter (A) Full view. (B) Proximal end feature sets. (C) Distal end functionalities. The easily maneuverable and torqueable imaging catheter enables the operator to steer and aim an 0.014-inch guidewire out the side port in any continuous orientation with better than 5-degree precision. (D) Cross-sectional cartoon representation of the catheter as a reference to the optical coherence tomography (OCT) images in Figure 5. Within the catheter cross-section, the imaging lumen and the indicator lumen are “static” or permanent features independent of the imager’s position. The guidewire lumen, guidewire ramp lumen, side port exit, and guidewire are all dynamic elements depending on the location of the guidewire as well as the longitudinal position of the OCT imager within the distal nosecone of the catheter.

**Figure 4:**
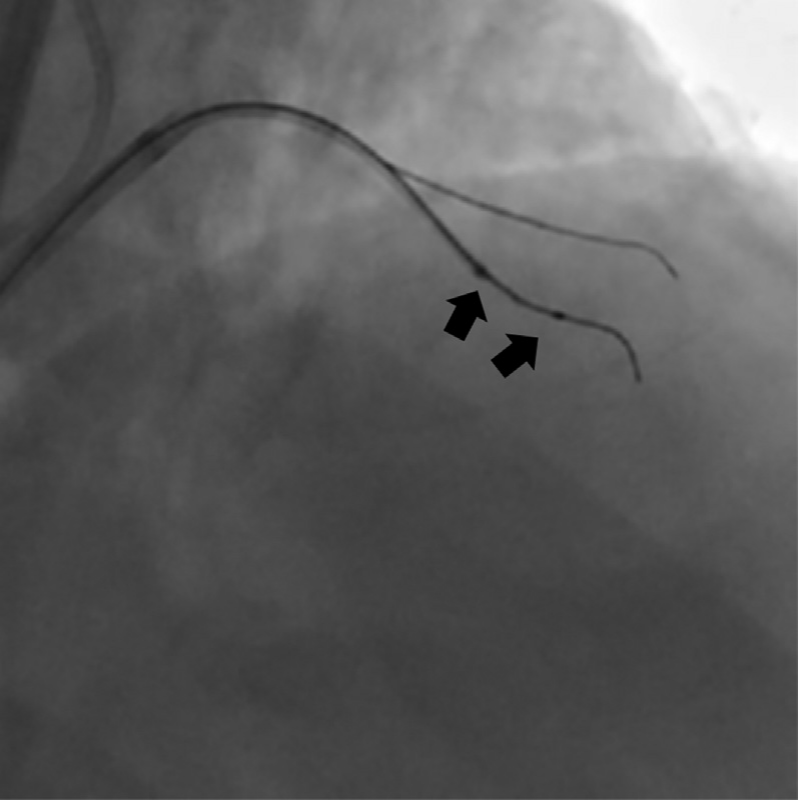
Angiographic View The operator proceeded to antegrade dissection and re-entry with the image-guided re-entry catheter (2 arrows pointing to markers) shown here approximately 1 cm proximal to the end of the guidewire and advancing toward the distal cap.

**Figure 5:**
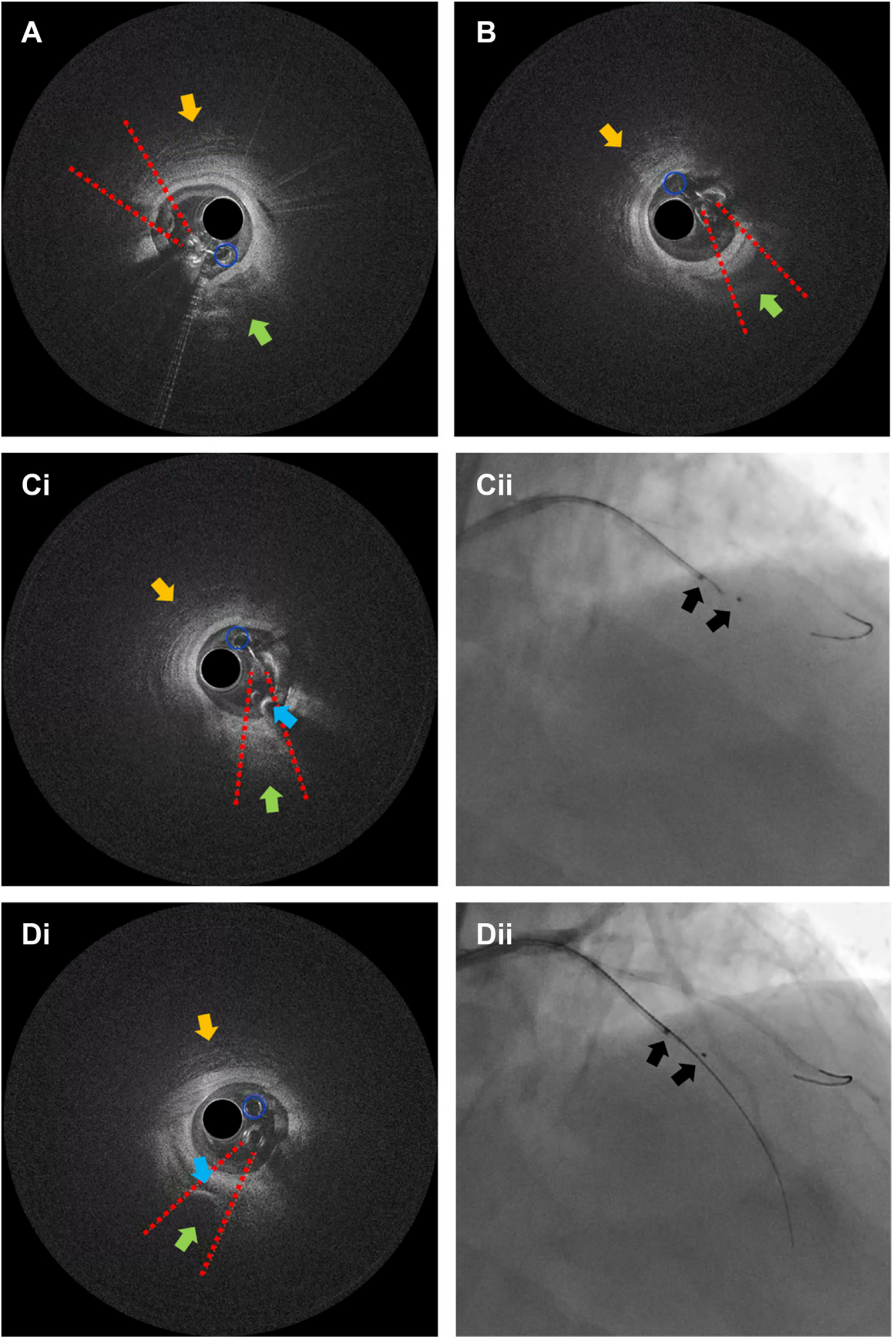
Optical Coherence Tomography Imaging View OCT was initiated with real-time ramp overlay augmentation (redline projections with an accompanying blue circle) enabled. The ramp overlay augmentation indicates the side port orientation from which guidewire advancement would be exiting in between the 2 redline projections of the overlay. The blue circle, which is part of the ramp overlay, provides the operator a reference to gauge the accuracy of software/AI inference of the ramp overlay graphics; the ramp overlay is most accurate when the blue circle is aligned with the indicator lumen (when they are concentric on top of each other, see Figure 3D for indicator lumen). If the blue circle is qualitatively offset against the indicator lumen, the ramp overlay including the 2 redline projections are offset to the side port exit accordingly by the same amount. In this view (A), which also corresponds to angiogram in Figure 4, the ramp overlay is pointing in the direction of the adventitia/perivascular structure (orange arrow pointing to fibrous/honeycomb layer structure from 10 to 1 o’clock), and the true lumen is on the opposing side (green arrow). In B, the catheter was torqued/rotated to redirect the ramp overlay augmentation away from the adventitia/perivascular structure, in the opposing direction with the overlay now in alignment with the true lumen (green arrow pointing to crescent dark shape at 5 o’clock). In this view, the true lumen of the LAD was seen pulsating in real time and in the recorded video clip (Video 4). (Ci) A hydrophilic-coated 20-gf guidewire was advanced through the side port, seen here with the guidewire (blue arrow pointing to bright crescent-shaped object) exiting the side port in between the 2 redline projections of the ramp overlay; The corresponding angiogram (Cii) shows the guidewire exiting the exit port; the hydrophilic-coated 20-gf guidewire was used to “stick and drive.” (Di) Optical coherence tomography (OCT) images confirmed re-entry, showing guidewire (blue arrow, bright crescent-shaped object) within the pulsating true lumen (green arrow). (Dii) Angiographic view confirming guidewire position in the true lumen. For video recording of OCT imaging, see Video 4.

**Figure 6:**
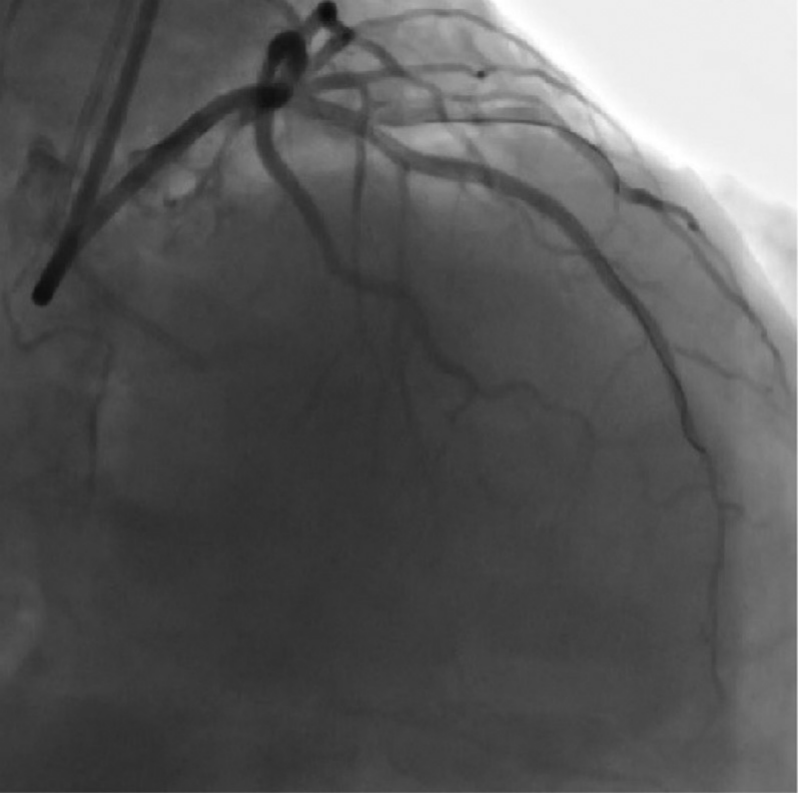
Angiographic View Full recanalization of the left anterior descending after stenting.

An antegrade wire escalation (AWE) strategy was initially used; however, a polymer-jacketed soft-tip guidewire (Gladius Mongo, Asahi Intecc) was noted to be in the extraplaque space (Figure 2, Video 2). Parallel wiring was also attempted with multiple guidewires, but this was unsuccessful in wiring the distal true lumen. We therefore proceeded with antegrade dissection and re-entry (ADR) using the novel image-guided re-entry catheter (The Acolyte, Simpson Interventions, Inc.) (Figures 3A to 3D). The catheter was advanced over a silicone-coated 12-gf guidewire (Miraclebros 12, Asahi Intecc) past the distal cap to the re-entry zone in the extraplaque space (Figure 4, Video 3). Retrograde contrast material injection showed poor distal target visualization caused by extraplaque hematoma formation. Using augmented real-time optical coherence tomography (OCT) image guidance, a pulsating artery with blood flow was visualized past the distal cap of the CTO from the extraplaque space (at 5 o’clock in the OCT image/clip) (Figure 5A, Video 4). The pulsating true lumen was observed as a temporally alternating optically scattering medium—blood flow from collateral arteries—occupying a cross-sectional area opposing the direction of the layered adventitia and perivascular structure, at the patient’s heart rate. The catheter was then torqued/rotated to redirect the exit ramp overlay away from the adventitia (10 to 1 o’clock in the OCT image/clip) and toward the true lumen at 5 o’clock in the OCT image/clip (Figure 5B, Video 4). The “exit ramp overlay” or “ramp overlay” is a graphical augmentation consisting of 2 redline projections and a blue circle reference that are updated in real time over the OCT images during the procedure. The redline projections of the ramp overlay represent the direction or trajectory of the guidewire exiting the ramp leading to a side port just proximal to the distal tip of the catheter; this provides real-time information to the operator to enable him/her to steer a guidewire in the direction of the true lumen for re-entry. A hydrophilic-coated 20-gf guidewire (Astato XS 20, Asahi Intecc) was then advanced through the dedicated re-entry port exit ramp (Figures 5Ci and 5Cii) to successfully re-enter the true lumen in the mid LAD. The guidewire position in the true lumen was confirmed by angiography and OCT imaging (Figures 5Di and 5Dii, Video 4). Figure 6 shows the final angiographic result after drug-eluting stent deployment (Video 5). The patient tolerated the procedure well without any periprocedural or in-hospital complications.

## Discussion

Anatomically complex cases of CTO often require leveraging the extraplaque or formerly “subintimal” space for successful CTO recanalization. The ADR technique is an important part of the hybrid approach to contemporary CTO percutaneous coronary intervention (PCI).^1^ However, despite the availability of dedicated re-entry devices, the success rate for ADR is around 50% to 60% in large registries.^2^^,^^3^ This is often due to large extraplaque hematoma formation leading to loss of distal vessel visualization, making re-entry challenging. Although the retrograde approach has led to an increase in overall CTO PCI success rates, it is associated with higher complication rates. In comparison to antegrade-only, retrograde approaches are associated with lower technical success rates and higher in-hospital rates of major adverse cardiac events, contrast volume, radiation dose, and procedure time.^4^^,^^5^

The study was approved by a research ethics committee. The patient provided signed ethics committee–approved informed consent. In this study, we demonstrate the use of the novel 4-F OCT image-guided re-entry catheter (Figure 3) in a first-in-human case of contemporary ADR that enables real-time high-resolution visualization with graphical augmentation, precision steering, and advancement of a guidewire for re-entry (Figure 5, Video 4). The OCT imaging element is situated/integrated within an imaging lumen of the re-entry catheter. The catheter is advanced over the wire to the intraplaque or extraplaque space depending on the position of the guidewire within the vessel. Aside from its high resolution enabling clear visualization of layered structures, plaque, and pulsating true lumen, it is worth noting that the utility of OCT in an occluded artery and extraplaque space was visually optimal, inasmuch as blood flow was minimal in the tight space occupied by a 4-F catheter, including when a subintimal hematoma was present. The image-guided re-entry catheter provided for minimal flushing (saline or contrast material) to remove blood (eg, optical scattering media) surrounding the imaging element; saline or contrast material flushed through the imaging element was re-routed to exit 10 millimeters proximal to the catheter tip, which displaced the blood outside the catheter adjacent to the imaging element in order to reduce the risk of hydrodissection. This eliminates the need for contrast or saline flush through the guiding catheter and therefore minimizes the propagation of hydraulic dissection. A 3-way stopcock was used with the flush port to maintain pressure from backflow, keeping blood away from the surrounding of the imaging element. In future embodiments, subintimal aspiration could further enhance the performance of this image-guided re-entry catheter. Contemporary ADR technique often involves using a stiff wire to “stick and drive” for re-entry into the true lumen. However, this is not always successful, especially in the setting of compressed true lumen from the extraplaque space hematoma. In such cases, a “stick and swap” technique is often used whereby multiple fenestrations are made with a sharp wire, followed by a polymer-jacketed wire to wire the true lumen. We followed the same principles for ADR with this device. If the “stick and drive” with the sharp wire was unsuccessful, our next step would have been to perform a “stick and swap,” for instance, with an Astato XS 20 (Asahi Intecc) followed by a Pilot 200 wire (Abbott Vascular).

The image-guided re-entry catheter is the first vascular imaging system to have “self-awareness” with real-time OCT-based augmentation, which provides orientation information of the catheter relative to vascular morphology (eg, true lumen, adventitia, perivascular structure, calcified nodule), enabling the user to directionally control the coronary guidewire for re-entry. This real-time image-guided re-entry helps overcome challenges posed by extraplaque hematoma formation and subsequent loss of distal vessel visualization. The high resolution provides clear morphologic details in conjunction with software augmentation for ease of use, representing a significant advancement over current device-based re-entry approaches and intravascular ultrasound–guided techniques. The Central Illustration describes the utility of the image-guided re-entry catheter during CTO PCI, with realtime augmentation to aid in re-directing a guidewire from the extraplaque space into the true lumen.

**Central Illustration:**
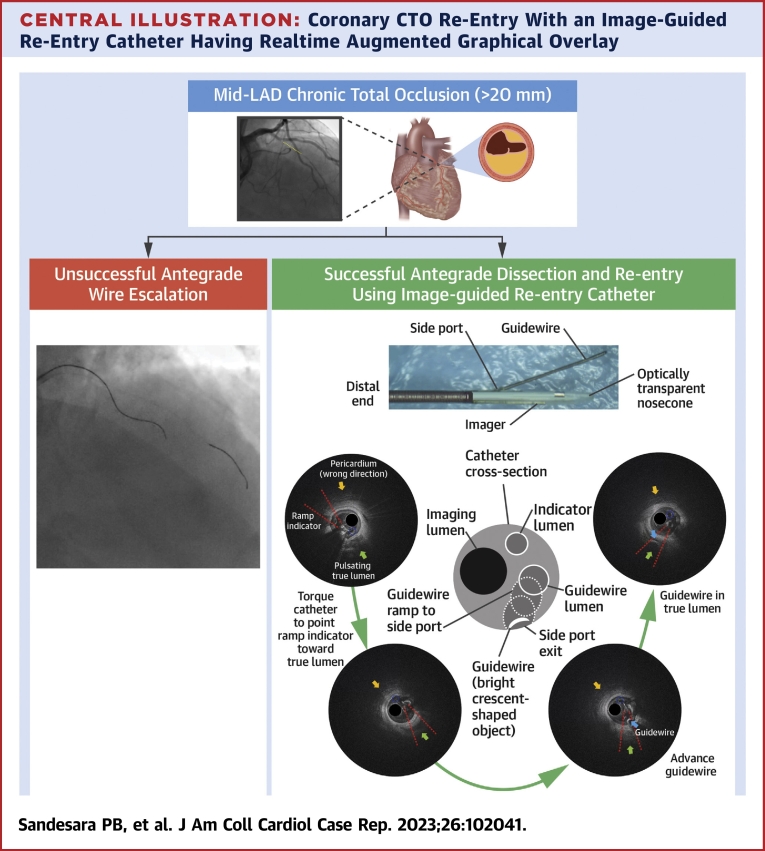
Coronary CTO Re-Entry With an Image-Guided Re-Entry Catheter Having Realtime Augmented Graphical Overlay

## Follow-Up

The patient did well after the procedure and did not experience any periprocedural or in-hospital adverse events. At the 30-day follow-up visit, he had experienced a significant improvement in symptoms and was free of angina.

## Conclusions

The success rates for contemporary ADR are suboptimal because of loss of distal vessel visualization as a result of extraplaque hematoma formation. The novel OCT-guided re-entry catheter is a promising tool to help overcome this barrier to successful ADR for CTO PCI.

## Funding Support and Author Disclosures

The real-time AI/software inference (eg, ramp overlay) is developed by and is the intellectual property of Simpson Interventions. This work was funded by Simpson Interventions, Inc. Drs Sandesara, Robertson, Ebner, Lombardi, Kandzari, and Hinohara and Ms Minarsch are consultants for Simpson Interventions. Drs Chan and Simpson and Mr Rowe are employees of Simpson Interventions.

## Appendix

Video 1 Angiography of a Mid-LAD CTO Angiography indicates occlusion length of >20 mm and calcification within the CTO segment (J-CTO score of 2).

Video 2 Unsuccessful Revascularization Attempt With Antegrade Wire Escalation Guidewire was noted to be in the extraplaque space after approximately 45-min of antegrade wire escalation attempt.

Video 3 Angiography Showing Deployment of Image-Guided Re-entry Catheter The catheter was advanced over a silicone-coated 12-gf guidewire past the distal cap to the re-entry zone in the extraplaque space.

Video 4 The Image-Guided Re-entry Catheter Featuring OCT With Real-Time Image Augmentation Demonstration of precision guidance, steering, and advancement of a hydrophilic-coated 20-gf guidewire. Recordings of ADR case by the image-guided re-entry catheter, resulting in successful re-entry and confirmed by both OCT and angiography. The catheter, as indicated by the ramp overlay with the redline projections, was initially oriented in the direction of the adventitia/perivascular structure. Advancing a guidewire would have resulted in perforation. The operator torqued and re-oriented the catheter to align the redline projections in the direction of the pulsating true lumen. The operator then proceeded to advance the guidewire. The guidewire appears as a crescent shape moving object as it was advanced from within the catheter, traversing the plaque from the extraplaque space, toward the pulsating true lumen between 4’ and 6’ o’clock over the duration of the video clip (from 2:03 to 2:44), in general agreement with the ramp overlay’s redline projections.

Video 5 LAD Successfully Revascularized Angiography following drug-eluting stent deployment.
